# Supplementary figures and images for: Detection of Cytosolic Ion Concentrations by the Trans-Golgi Network/Early Endosome is Important for Salt Tolerance
Source: bioRxiv. 2025 Aug 17:2025.08.13.670069. Preprint. [Version 1] doi: 10.1101/2025.08.13.670069 (PMC12363952; doi:10.1101/2025.08.13.670069)

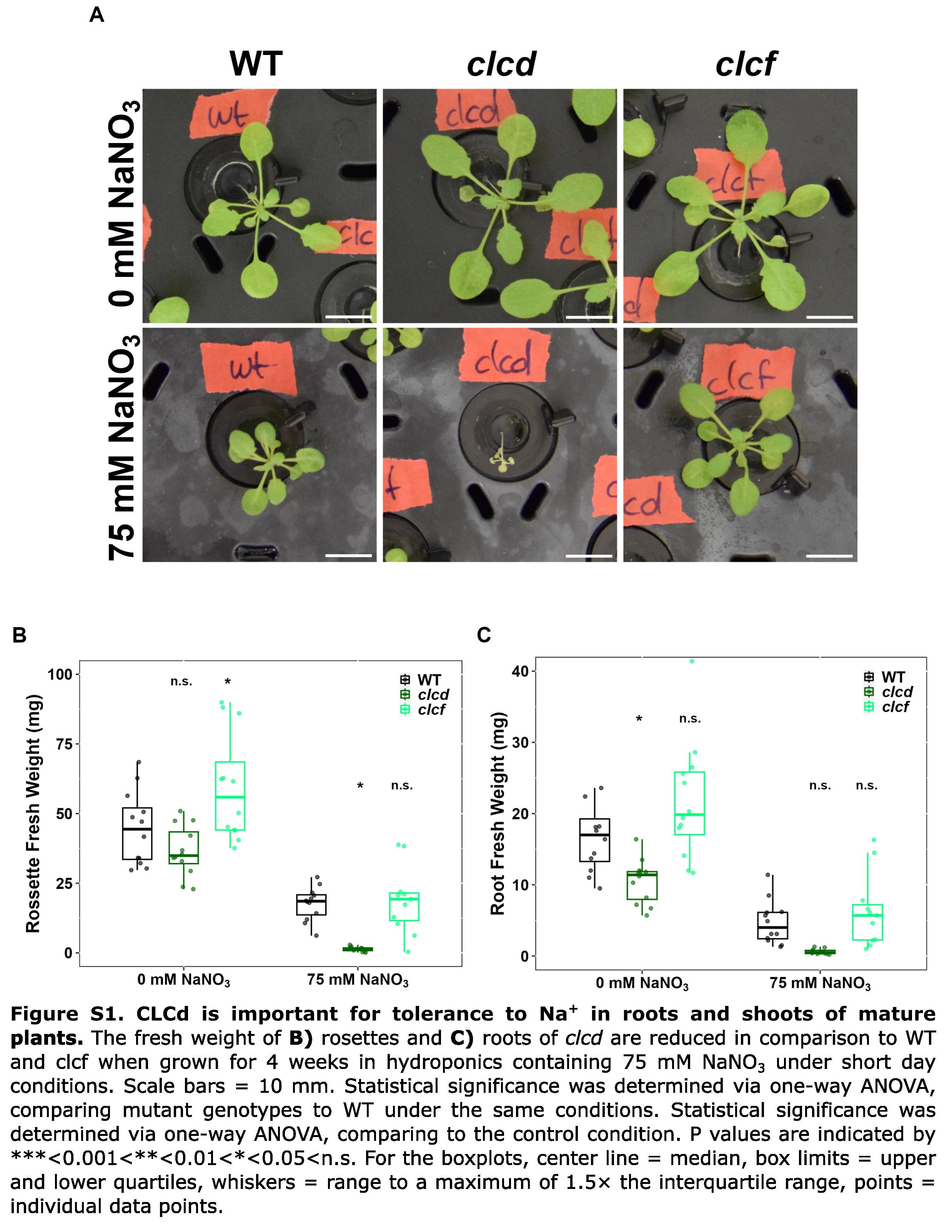

Supplement: Supplement 1 — Figure S1. CLCd is important for tolerance to Na+ in roots and shoots of mature plants The fresh weight of B) rosettes and C) roots of clcd are reduced in comparison to WT and clcf when grown for 4 weeks in hydroponics containing 75 mM NaNO3 under short day conditions. Scale bars = 10 mm. Statistical significance was determined via one-way ANOVA, comparing mutant genotypes to WT under the same conditions. Statistical significance was determined via one-way ANOVA, comparing to the control condition. P values are indicated by ***<0.001<**<0.01<*<0.05<n.s. For the boxplots, center line = median, box limits = upper and lower quartiles, whiskers = range to a maximum of 1.5× the interquartile range, points = individual data points. [file media-1.jpg]

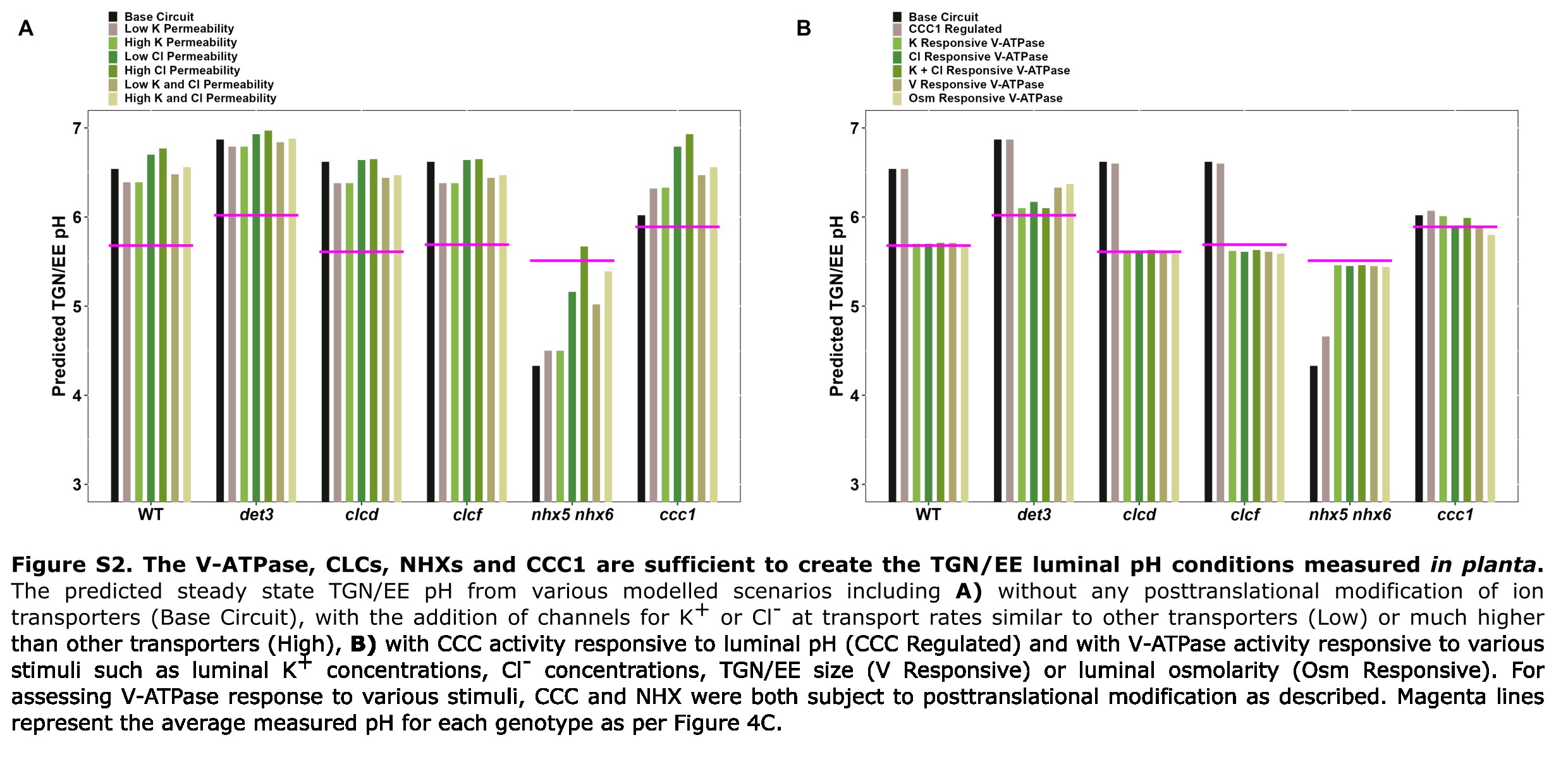

Supplement: Supplement 2 — Figure S2. The V-ATPase, CLCs, NHXs and CCC1 are sufficient to create the TGN/EE luminal pH conditions measured in planta. The predicted steady state TGN/EE pH from various modelled scenarios including A) without any posttranslational modification of ion transporters (Base Circuit), with the addition of channels for K+ or Cl− at transport rates similar to other transporters (Low) or much higher than other transporters (High), B) with CCC activity responsive to luminal pH (CCC Regulated) and with V-ATPase activity responsive to various stimuli such as luminal K+ concentrations, Cl− concentrations, TGN/EE size (V Responsive) or luminal osmolarity (Osm Responsive). For assessing V-ATPase response to various stimuli, CCC and NHX were both subject to posttranslational modification as described. Magenta lines represent the average measured pH for each genotype as per Figure 4C. [file media-2.jpg]

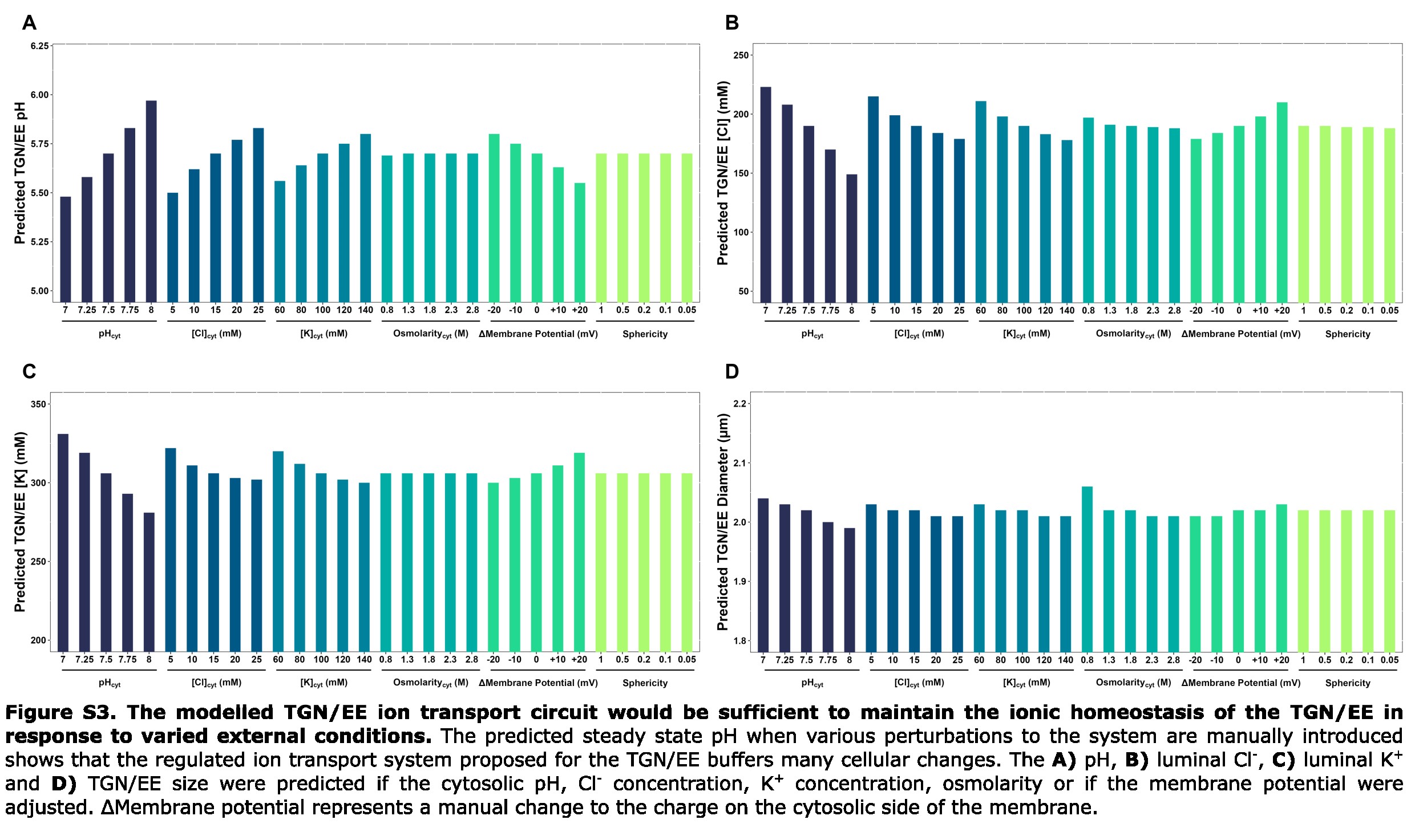

Supplement: Supplement 3 — Figure S3. The modelled TGN/EE ion transport circuit would be sufficient to maintain the ionic homeostasis of the TGN/EE in response to varied external conditions. The predicted steady state pH when various perturbations to the system are manually introduced shows that the regulated ion transport system proposed for the TGN/EE buffers many cellular changes. The A) pH, B) luminal Cl−, C) luminal K+ and D) TGN/EE size were predicted if the cytosolic pH, Cl− concentration, K+ concentration, osmolarity or if the membrane potential were adjusted. ΔMembrane potential represents a manual change to the charge on the cytosolic side of the membrane. [file media-3.jpg]

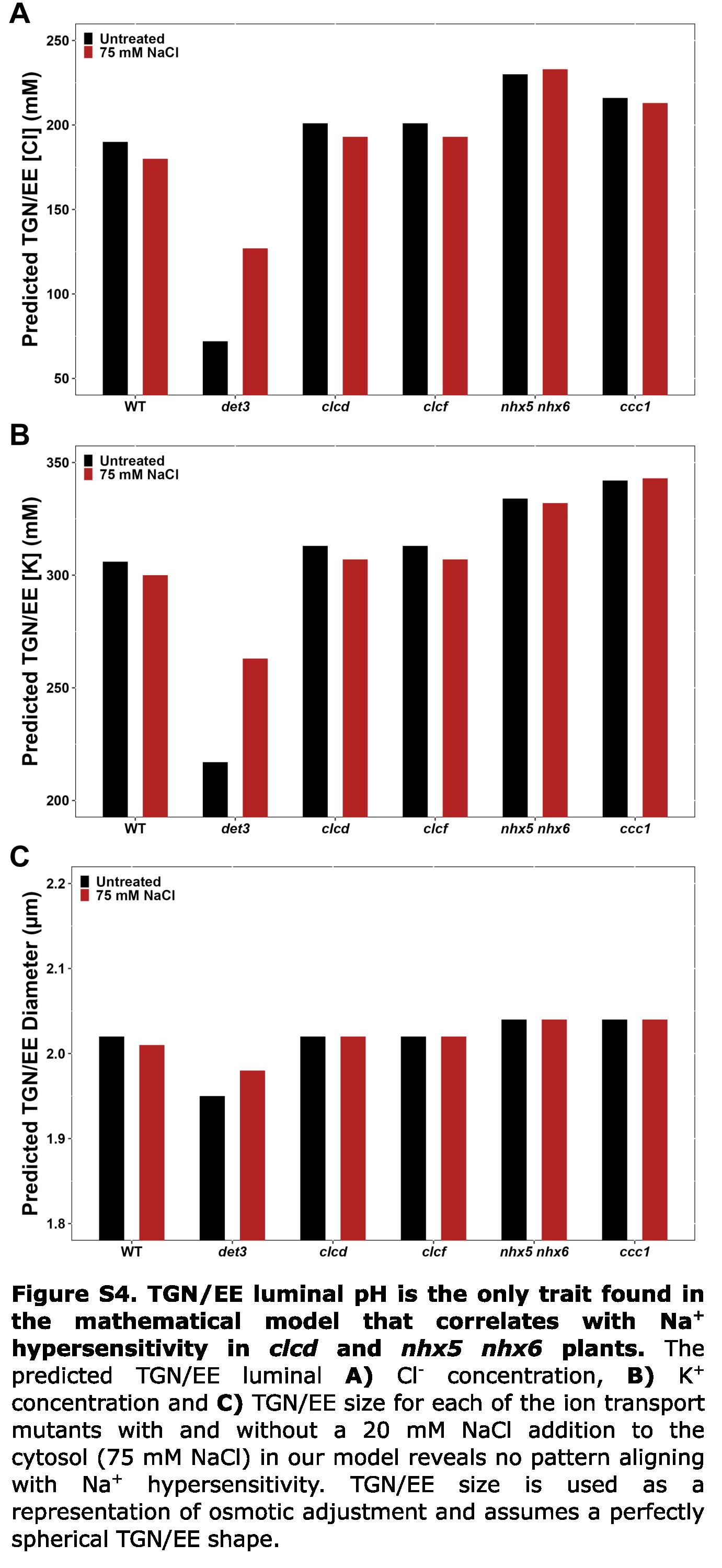

Supplement: Supplement 4 — Figure S4. TGN/EE luminal pH is the only trait found in the mathematical model that correlates with Na+ hypersensitivity in clcd and nhx5 nhx6 plants. The predicted TGN/EE luminal A) Cl− concentration, B) K+ concentration and C) TGN/EE size for each of the ion transport mutants with and without a 20 mM NaCl addition to the cytosol (75 mM NaCl) in our model reveals no pattern aligning with Na+ hypersensitivity. TGN/EE size is used as a representation of osmotic adjustment and assumes a perfectly spherical TGN/EE shape. [file media-4.jpg]

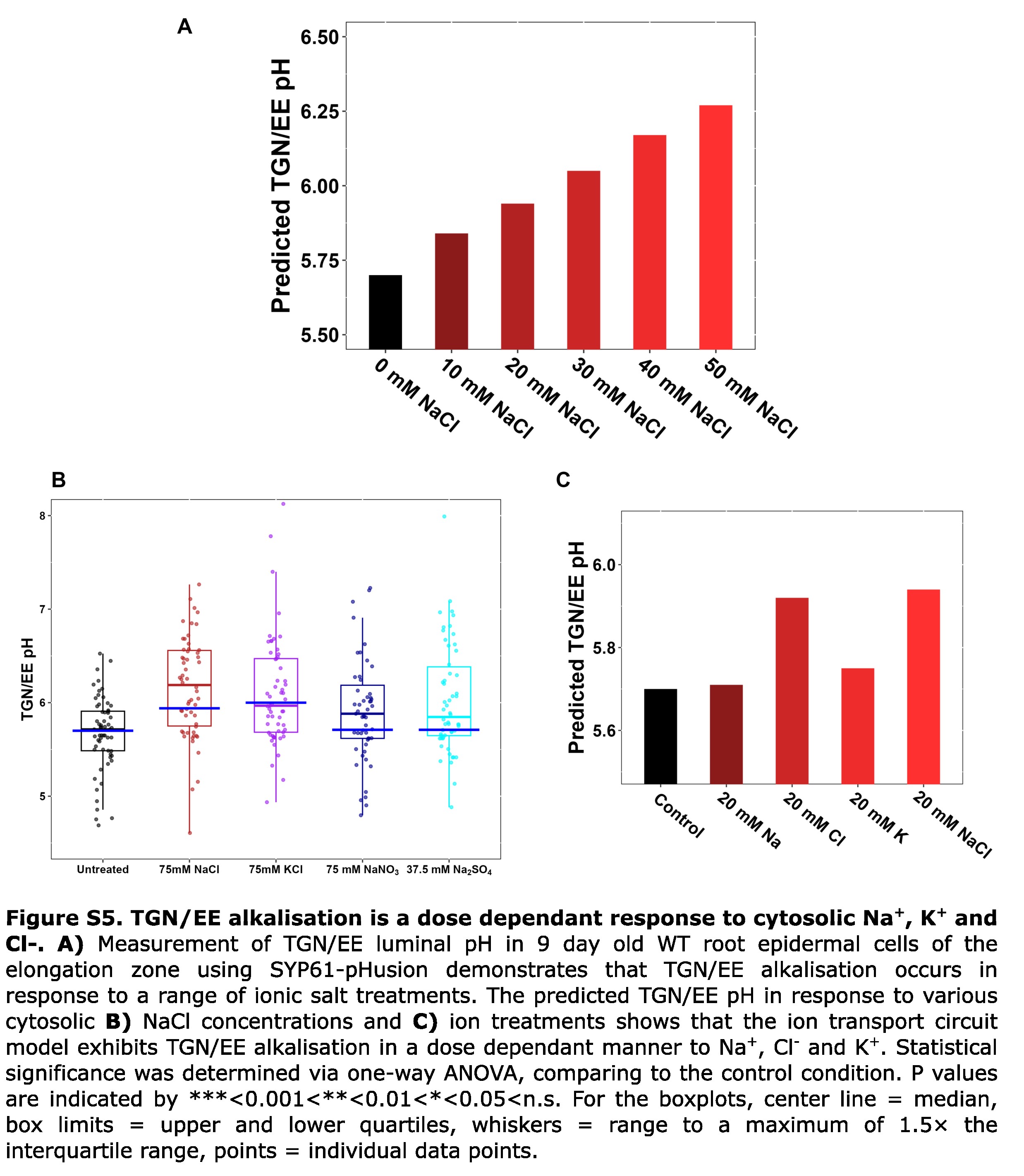

Supplement: Supplement 5 — Figure S5. TGN/EE alkalisation is a dose dependant response to cytosolic Na+, K+ and Cl−. A) Measurement of TGN/EE luminal pH in 9 day old WT root epidermal cells of the elongation zone using SYP61-pHusion demonstrates that TGN/EE alkalisation occurs in response to a range of ionic salt treatments. The predicted TGN/EE pH in response to various cytosolic B) NaCl concentrations and C) ion treatments shows that the ion transport circuit model exhibits TGN/EE alkalisation in a dose dependant manner to Na+, Cl− and K+. Statistical significance was determined via one-way ANOVA, comparing to the control condition. P values are indicated by ***<0.001<**<0.01<*<0.05<n.s. For the boxplots, center line = median, box limits = upper and lower quartiles, whiskers = range to a maximum of 1.5× the interquartile range, points = individual data points. [file media-5.jpg]
